# Case Report of Long QT Syndrome in a Patient With Syncope

**DOI:** 10.1002/kjm2.70065

**Published:** 2025-06-17

**Authors:** Chun‐Yu Chen, Rui‐Xian Wang, Ken‐Pen Weng, Shih‐Ming Huang

**Affiliations:** ^1^ Department of Pediatrics Chi Mei Medical Center Tainan Taiwan; ^2^ Department of General Education Center Chia‐Nan University of Pharmacy and Science Tainan Taiwan; ^3^ Department of Pediatrics Kaohsiung Veterans General Hospital Kaohsiung Taiwan; ^4^ Department of Pediatrics Kaohsiung Municipal United Hospital Kaohsiung Taiwan

Long QT syndrome (LQTS) is an abnormal prolongation of the QT interval, as determined using electrocardiography, that can cause life‐threatening ventricular arrhythmias. Herein we present the case of a woman with syncope and ventricular arrhythmia as well as KCNH2 mutation, which was identified through a genetic study.

A 17‐year‐old female patient experienced syncope but quickly recovered while exercising. Although she experienced no prodrome (i.e., palpitation or chest pain), she visited our out‐patient department because of the syncope. Physical examination revealed an irregular heartbeat without a murmur. Initial 12‐lead electrocardiography revealed QT prolongation (QTc 702 ms) and ventricular premature contractions. Echocardiography revealed mild mitral regurgitation and preserved left ventricle ejection fraction without structural abnormalities. Her initial hs‐troponin‐I level was 83.1 pg/mL, which was normal during follow‐up. Her thyroid function and electrolyte levels were normal. On admission day 1, her heart rhythm occasionally exhibited ventricular tachycardia, ventricular fibrillation, and Torsades de pointes. Because the rhythm repeatedly returned to sinus rhythm without intervention or medication, we continued to closely observe her arrhythmia. Subsequent monitoring with 24‐h Holter electrocardiography revealed frequent ventricular premature contractions (i.e., 672 couplet, 43 bigeminy, 6 triplet, and 4 nonsustained ventricular tachycardia rhythms). The only symptoms the patient mentioned were dizziness and mild chest pain. On admission day 2, we initiated propranolol administration to reduce the risk of a cardiac event. The arrhythmia resolved, and an exercise test did not provoke any ventricular arrhythmia. Considering her family history—her grandfather experienced cardiac arrest with an unknown cause 10 years prior—congenital LQTS was suspected. Assessment revealed a Schwartz score offive (electrocardiogram > 480 ms, three points; syncope with stress, two points), which suggested LQTS. A genetic study was performed in the laboratory of a center hospital. The genetic study involved next‐generation sequencing, step 2 whole exome sequencing, and step 3 whole genome sequencing. The results revealed a KCNH2 mutation at chromosome 7 with nucleotide change (c.1755G > T heterozygous) and amino acid change (p.Trp585Cys), which is considered pathogenic in ClinVar. The family denied genetic screening and consultation for personal reasons. The patient was prescribed atenolol and then discharged and did not experience another ventricular arrhythmia event during follow‐up Figure 1.

**FIGURE 1:**
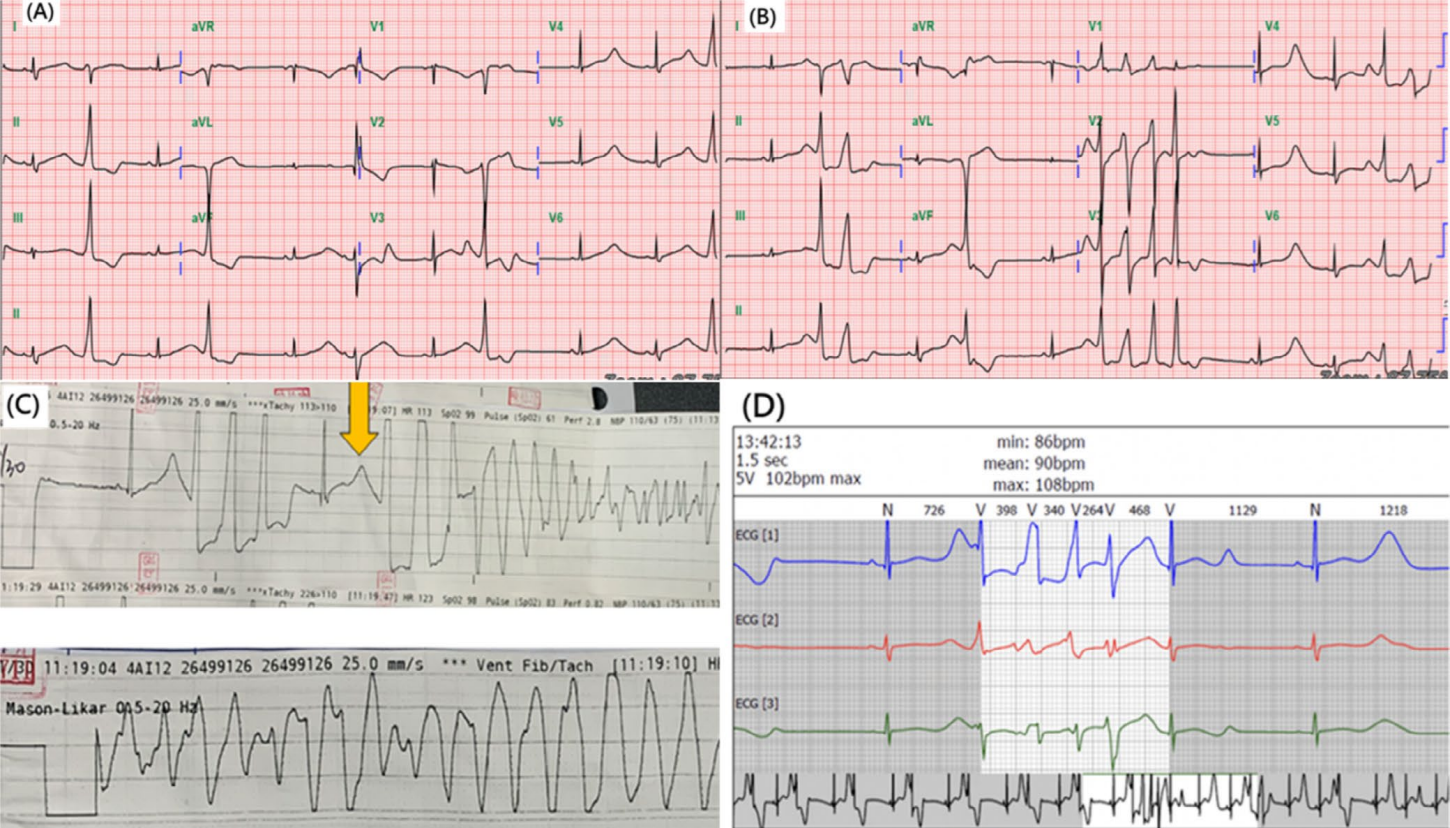
(A) Initial EKG revealing prolonged QTc interval of 685 ms accompanied by ventricular premature complexes. (B) Subsequent EKG revealing prolonged QTc with a short run of ventricular tachycardia. (C) Long EKG strip indicating R on T with TdP. (D) Holter revealing nonsustained VT.

The patient discussed herein was determined to have LQTS on the basis of clinical history, electrocardiography, and a genetic study. LQTS is a heterogeneous disorder involving various genetic mutations that affect cardiac ion channels, leading to delayed ventricular repolarization. The patient in this study had a mutation in the KCNH2 gene, which is consistent with a diagnosis of type 2 LQTS, a life‐threatening disorder caused by a rapid delayed rectifier K+ current. According to the literature, auditory stimuli are common triggers for life‐threatening arrhythmias in LQT2, and seizures frequently occur following cardiac syncope [1]. In our case, the patient did not experience a seizure, and the syncope was triggered by exercise.

Research indicates that a beta blocker should be used as the first‐line therapy in LQTS with wide‐complex tachycardia [2]. In the present case, antiarrhythmic drugs and inotropic agents such as amiodarone, epinephrine, and isoproterenol were not required. Notably, amiodarone should be used carefully in cases of LQTS because it can lead to QT prolongation, hypotension, and bradycardia. Amiodarone has a long half‐life and can delay the resolution of adverse effects, with effects potentially occurring after the discontinuation of the medication. Some researchers recommend that amiodarone be avoided in patients with LQTS, suggesting that its use may contribute to refractory ventricular tachycardia and Torsades de pointes [3]. Furthermore, epinephrine should be used with caution in patients with LQTS because it may act as a provocative stimulus and is used in stress testing [4]. Isoproterenol, a nonselective beta agonist, is often used to increase heart rate but is contraindicated in congenital LQTS because it paradoxically lengthens the QT interval [5]. Overdrive pacing is the final choice in LQTS, and defibrillation for ventricular tachycardia and ventricular fibrillation may not be useful. Furthermore, left cardiac sympathetic denervation and internal cardioverter defibrillators may be considered, but only in severe cases. The case reported herein has clinical implications because it indicates that early use of oral beta blockers can be effective in cases of LQTS with Torsades de pointes, and immediate cardioversion may not be necessary if the ventricular arrhythmia is self‐terminating.

## Conflicts of Interest

The authors declare no conflicts of interest.

## Data Availability

Data sharing is not applicable to this article as no new data were created or analyzed in this study.
